# Something old, something new and something borrowed: emerging paradigm of insulin-like growth factor type 1 receptor (IGF-1R) signaling regulation

**DOI:** 10.1007/s00018-013-1514-y

**Published:** 2013-11-26

**Authors:** Leonard Girnita, Claire Worrall, Shin-Ichiro Takahashi, Stefan Seregard, Ada Girnita

**Affiliations:** 1 Department of Oncology and Pathology, Cancer Center Karolinska, CCK R8:04, Karolinska Institute, Karolinska University Hospital, 171 76 Stockholm, Sweden; 2 Departments of Animal Sciences and Applied Biological Chemistry, Graduate School of Agriculture and Life Sciences, The University of Tokyo, Tokyo, Japan; 3 Ophthalmic Pathology and Oncology Service, St Erik’s Eye Hospital and Karolinska Institute, Stockholm, Sweden; 4 Dermatology Department, Karolinska University Hospital, 171 76 Stockholm, Sweden

**Keywords:** IGF-1R, GRKs, IRS, Beta-arrestins, Serine phosphorylation, Cancer, Ubiquitination, GPCR, RTK

## Abstract

The insulin-like growth factor type 1 receptor (IGF-1R) plays a key role in the development and progression of cancer; however, therapeutics targeting it have had disappointing results in the clinic. As a receptor tyrosine kinase (RTK), IGF-1R is traditionally described as an ON/OFF system, with ligand stabilizing the ON state and exclusive kinase-dependent signaling activation. Newly added to the traditional model, ubiquitin-mediated receptor downregulation and degradation was originally described as a response to ligand/receptor interaction and thus inseparable from kinase signaling activation. Yet, the classical model has proven over-simplified and insufficient to explain experimental evidence accumulated over the last decade, including kinase-independent signaling, unbalanced signaling, or dissociation between signaling and receptor downregulation. Based on the recent findings that IGF-1R “borrows” components of G-protein coupled receptor (GPCR) signaling, including β-arrestins and G-protein-related kinases, we discuss the emerging paradigm for the IGF-1R as a functional RTK/GPCR hybrid, which integrates the kinase signaling with the IGF-1R canonical GPCR characteristics. The contradictions to the classical IGF-1R signaling concept as well as the design of anti-IGF-1R therapeutics treatment are considered in the light of this paradigm shift and we advocate recognition of IGF-1R as a valid target for cancer treatment.

## Introduction

Transmission of extracellular signals across the plasma membrane by receptors is a fundamental, evolutionary conserved cellular process. These signals are in part generated by specialized plasma membrane receptors, grouped into different families based on their specific structural characteristics. Although different receptor families utilize common intracellular signaling-proteins and activate common signaling pathways, each cell surface receptor family leads to specific biological outcomes in the cell [1].

The receptor tyrosine kinases (RTKs) represent one major cell surface receptor family, containing around 60 members, subdivided into at least 13 receptor families [2, 3]. The RTKs are defined by the presence of an intracellular tyrosine kinase domain and typically a large glycosylated extracellular ligand binding domain, separated by a single transmembrane pass [4]. Traditionally defined by their ligands and hence ligand binding domains, the cytoplasmic kinase regions, juxtamembrane domain and carboxyl (C)-terminal tail also differ significantly among the individual receptors.

The canonical, binary model still in use today, describes the RTKs as having an OFF/ON mechanism. The agonist stabilizes the “ON” state through autophosphorylation of tyrosine residues within the kinase domain, followed by exponential increase in its kinase activity and subsequent activation of the intra-cellular signaling pathways [1]. In most cases, the ligand-induced activation of the kinase domain is mediated by receptor oligomerization (for reviews, see [4–6]). This event favors interactions between cytoplasmic kinase-partners and induces kinase transactivation. Dimerization can take place between two identical receptors (homodimerization), between different members of the same receptor family, or, in some cases, between a receptor and an accessory protein (heterodimerization) [6–9]. How ligands bind to receptors and induce oligomerization seems specific for each class of RTK [7, 10].

Over the last decades, RTKs have received particular attention, not only as essential regulators of normal cellular processes but also as key factors involved in the development and progression of human cancers. From the first discovery of a mutated RTK in cancer in 1984 [11], a huge amount of information about aberrant RTK signaling in cancer has built up, leading to incontestable recognition of various forms of RTK hyper-activation in cancer: gene amplification, overexpression, mutation, or autocrine growth factor loops that are responsible for the cancer-promoting potential of RTKs [12].

Among RTKs, the insulin-like growth factor type-1 receptor (IGF-1R) is one of the most important players in cancer development. The fundamental evidence for this is the demonstration that IGF-1R knock-out mouse embryonic cells are refractory to transformation by several oncogenes, viruses, or over-expression of other RTKs [13]. Subsequently, IGF-1R and its natural ligands were demonstrated to regulate multiple cellular functions essential for the malignant phenotype including cellular proliferation, survival, anchorage-independent growth, tumor neovascularization, migration, invasion, and metastasis [14–17]. Confirming this critical role, in preclinical settings, a large amount of experimental data clearly demonstrates that inhibition of IGF-1R would be beneficial for cancer treatment [18–24]. In vivo and in vitro studies using IGF-1R antibodies, small molecule inhibitors, and antisense technology have shown that IGF-1R is functionally essential for tumor cell growth and proliferation in most if not all forms of cancer [23, 25–28]. On the other hand, in clinical settings, no clear mechanism of aberrant IGF-1R could be recognized: IGF-1 or IGF-1R over-expression is not a general rule [17, 29], the receptor does not show intrinsic receptor abnormalities [30], and therefore other regulatory pathways and quantitative changes are likely to be involved and have to be considered.

This review will follow the development of our understanding of IGF-1R signaling and relate it to the models considered when different IGF-1R-targeting strategies are designed, in particular with regards to cancer therapy.

## Something old: classical signaling pathways through IGF-1R phosphorylation

The IGF family includes ligands, receptors, and IGF-binding-proteins (IGFBPs). Classically, there are three ligands (insulin, IGF-1, and IGF-2), three cell surface receptors [insulin receptor (IR), IGF-1R, and IGF-2R] and at least seven IGFBPs modulating the biological activity of the growth factors [31–33].

Besides these archetypal members, more recent work has identified other proteins as potential members of the IGF family: the antimicrobial peptide LL-37 as ligand [34], the orphan insulin-receptor-related receptor (IRR) [35], the insulin-IGF-1R hybrid receptor [36, 37], and a growing number of IGFBPs [38].

**Fig. 1:**
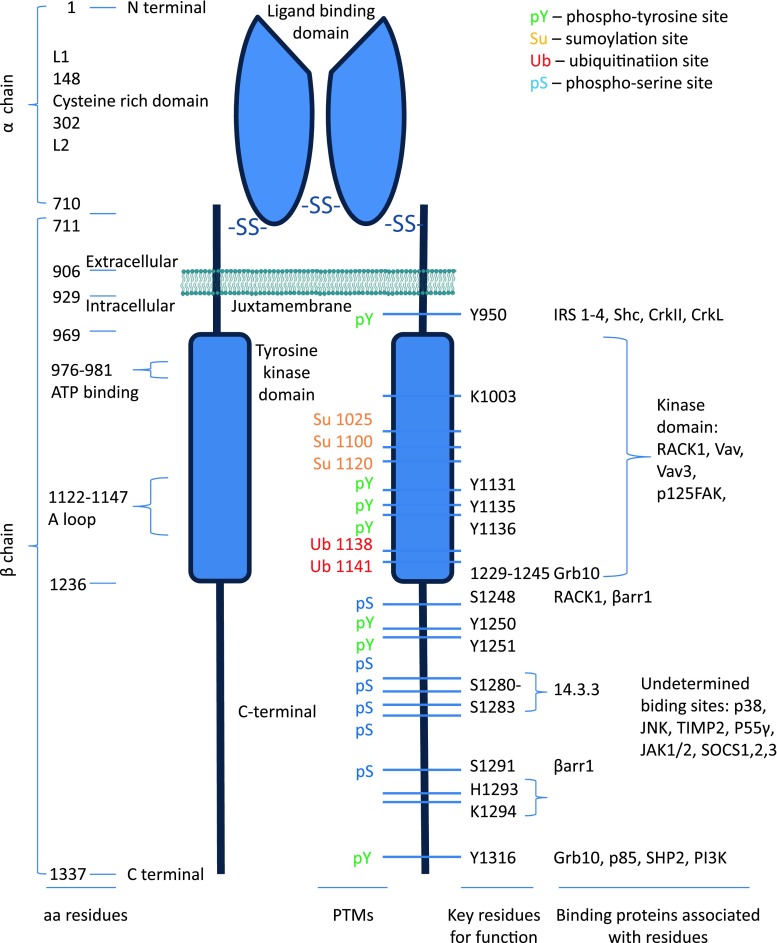
IGF-1R structure–function relationship. The map of the IGF-1R is annotated with the numbered aa residues. The key residues as determining binding of substrates/adaptor proteins are *highlighted* and linked with the binding partners. The known sites of posttranslational modifications (PTMs) within the β-subunit are indicated

Unlike other RTKs, the IR and IGF-1R exist as preformed dimers (Fig. 1). The IGF-1R has a membrane spanning tetrameric structure. It is synthesized as a single chain α-β pro-receptor which is processed by proteolysis and glycosylation [33, 39]. In mature, functional form, it consists of two identical extra cellular α-subunits and two identical β-subunits, all linked by disulfide bridges. The β-chain contains an extra cellular domain, a transmembrane domain, and a kinase-containing intracellular domain [39, 40] (Fig. 1). On the whole, there is high homology (70 %) between the IGF-1R and the IR amino acid sequences [40, 41].

The α-subunit contains 710 amino acids (aa 1–710) and has in its structure two homologous domains (L1 and L2) separated by a cysteine-rich domain (48 %) containing 25 or 27 cysteines, in three repeating units [33]. The cysteine-rich domain (aa 148–302) is responsible for the ligand binding and is also conserved in the IR [42–46]. The spanning plasma membrane β-subunit contains 627 amino acid residues (aa 711–1337), distributed among the extra cellular domain (196 aa), the transmembrane domain (aa 906–929), and the intracellular portion of the β-subunit, which itself is subdivided into three domains: a juxtamembrane domain, the tyrosine kinase (TK) domain, and C-terminal domain/tail. The juxtamembrane domain contains an NPXY motif, which may be important for receptor internalization [47–50]. The catalytic region of IGF-1R contains the ATP binding motif (GXGXXG) at positions 976–981, and a catalytic lysine in position 1003, which is critical for the Mg-ATP binding [51]. Within the TK domain, a cluster of three tyrosines, located at positions 1131, 1135, and 1136, is critical for receptor autophosphorylation [41]. The C-terminus of the IGF-1R (roughly the last 100 amino acids) contains several regulatory elements essential for IGF-1R function [52] (Table 1; Fig. 1).

**Table 1:** IGF-1R structure–function relationship

Residues	Binding partners	Functions
V922		M [267]
Y943		I [50]
Y950	IRS-1/2/3/4 [268–271], Shc [268, 269, 272], CrkII, CrkL [82, 273, 274]	I [49, 50], M, T [89, 275–278], pY
Y957		I [50]
Kinase domain 969–1236	RACK1 [87], Vav [80], Vav3 [81], p125FAK [83]	
976–981		ATP binding site, A [89]
K1003		A, M, T, K [89, 279]
K1025, K1100, K1120		Su [151]
K1081		[280]
G1125		K [281]
K1138, K1141		Ub [141]
Y1131, Y1135, Y1136		Auto-phosphorylation, M, T, K [89, 234, 276, 282, 283], pY
W1173		A, T [284]
Y1221		K [276]
1229–1245	Grb10 [78]	
S1248	RACK1 [88], βarr1 [124, 134]	pS
Y1250		T [128], M/I, A [89, 276, 285]
Y1251		I [286], K [276], A, T [89]
S1252		pS, I [280]
S1272	14.3.3 [287]	
S1280–S1283	14.3.3 [268, 288, 289]	T [290, 291]
S1291	βarr1 [124, 134]	pS
H1293–K1294		A [89, 292]
F1310		A [277]
Y1316	Grb10 [79], p85 [293], SHP2 [84, 145], PI3K [63]	[89, 292]
Undetermined	p38, JNK [294], TIMP2 [295], SOCS1/2 [86], SOCS3 [296], p55γ [297], JAK1/2 [298]	

The key residues for posttranslational modifications, protein–protein interaction and their functional outcome as determined by mutation-analysis are summarized

*K* kinase activity, *I* internalisation, *M* mitogenic, *T* transforming, *A* anti-apoptotic, *M/I* migration/invasion, *Su* Sumoylated residue, *Ub* ubiquitinated residue, *pY* phosphotyrosine, *pS* phosphoserine

The TK domain is highly homologous to that of the IR (84 %), the juxtamembrane domain shares 61 % of homology with the IR, whereas the C-terminal domain shares only 44 % [40]. Despite this high degree of homology, it is largely accepted that the two receptors have distinct biological roles. The IR is known to be a key regulator of physiological processes such as glucose transport and biosynthesis of glycogen and fat [53], whereas the IGF-1R is a potent regulator of cell growth, proliferation, and differentiation [54, 55]. The structure–function relationship of the IGF-1R has been extensively investigated, with mutational analysis revealing residues crucial for the binding of signaling or adaptor proteins (Table 1; Fig. 1) or particular downstream bioactivities (Table 1).

### IGF-1R tyrosine kinase activation

**Fig. 2:**
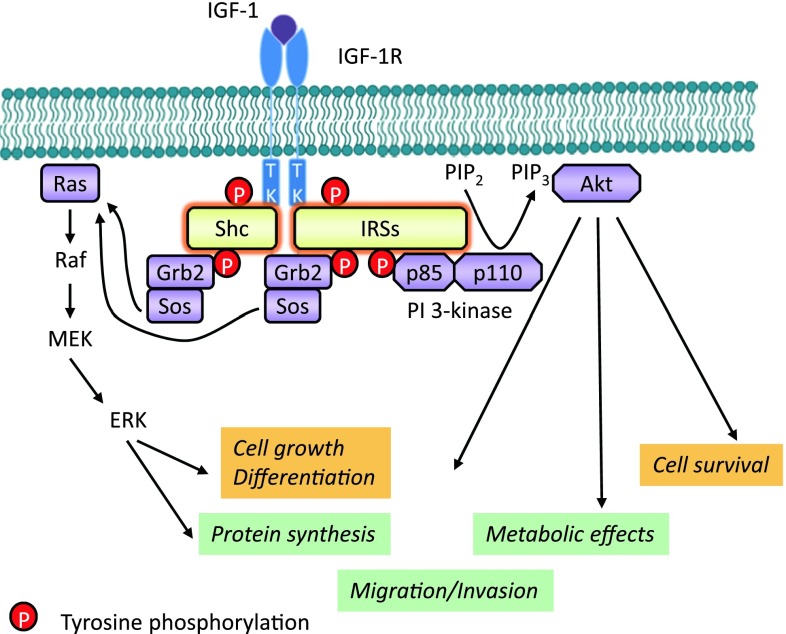
IGF-1R kinase-dependent signaling pathways. IGF-1 (or IGF-2) binding to the IGF-1R promotes intrinsic tyrosine kinase activity and auto-phosphorylation. Activated receptor can recruit and phosphorylate substrates such as IRS and Shc. Tyrosine phosphorylation of IRS and Shc proteins leads to binding of signaling molecules such as Grb2 and PI 3-kinase. These associations induce downstream signaling activation, primarily through the MAPK and PI3K pathways coordinating downstream IGFs bioactivities

According to the classical model, IGF-1/2 binding induces a conformational change in the preformed dimeric receptor, leading to activation of the RTK [6]. In the unphosphorylated state, the receptor catalytic activity is very low due to the inhibitory conformation of a specific domain in the kinase region, which interferes with ATP-binding and tyrosine phosphorylation. This domain, known as the activation loop (A-loop), behaves as a pseudosubstrate that blocks the active site [56]. The A-loop contains the critical tyrosine (Tyr) residues 1131, 1135, and 1136 and the activation of intrinsic protein kinase activity results in the autophosphorylation of them [56] (Fig. 1). Tyr 1135 (being the first tyrosine to be phosphorylated) in the A-loop is bound in *cis* position in the active site, thus preventing substrate access while simultaneously occluding the ATP binding site. The kinase activity is at a low basal level, but sufficient to induce trans-autophosphorylation, once stimulated. After ligand binding, the three tyrosines of the A-loop are *trans*-phosphorylated by the dimeric subunit partner. Phosphorylation of Tyr 1135 and Tyr 1131 destabilizes the auto-inhibitory conformation of the A-loop, whereas phosphorylation of Tyr 1136 and, to a lesser extent, Tyr 1135 stabilizes the catalytically optimized conformation of it [56]. Autophosphorylation of Tyr 1135 is necessary but not sufficient to destabilize the autoinhibitory A-loop conformation; full destabilization also requires autophosphorylation of Tyr 1131. Autophosphorylation also occurs outside the kinase domain and creates docking sites for downstream signal transduction molecules. According to the classical model, the two main downstream signaling pathways activated by IGF-1R kinase are the MAPK and PI3K pathways (Fig. 2).

### Intracellular substrates of the IGF-1R tyrosine kinase

The docking sites outside the kinase domain bind signal transduction molecules and adaptor proteins to activate downstream signaling pathways. One important docking site of the IGF-1R to a substrate is the NPXY950 motif in the juxtamembrane domain. This motif is recognized by the phosphotyrosine binding (PTB) domain that the insulin receptor substrates (IRSs) and the src homology 2 (SH2)-containing-protein (Shc) possess (Figs. 1, 2). Extensive mutation analysis exposed the specific residues involved in binding these substrates as indicated in Fig. 1 and Table 1.

After phosphorylation of the tyrosine residues of the β-subunit of the IGF-1R, the first molecule that reaches full binding in 1–2 min is the IRSs. The IRS protein family consists of four proteins from IRS1 to IRS4. Among them, IRS1 and IRS2 are well known to play important roles in mediating metabolic effects of the IGFs as well as their cell growth and differentiation activities. Each IRS possesses high homology regions: a pleckstrin homology domain (PH) and a PTB domain at the N-terminal region. These regions are thought to be important for interaction with cell surface receptors. The C-terminal region of IRS proteins is poorly conserved, suggesting that this region mediates the different biological activities of each IRS. It also has a C-terminal domain with multiple potential phosphorylation sites (more than 20 in IRS1/2/4) that interact with high affinity to SH2 domain-containing-proteins in a manner dependent on the specific phospho-tyrosine motif (YXXX) involved [57]. The SH2 domain-containing-proteins include a p85 regulatory subunit of phosphatidylinositol 3-kinase (PI3K) class I, growth-factor-receptor-bound protein 2 (Grb2), SH-PTP2 (a tyrosine phosphatase), and other adaptor proteins like Crk and Nck. IRS1, as a docking-protein, has been involved in interactions with multiple molecules secondary to IGF-1R activation, and among them β1 integrins seem to have an important role in cell adhesion to laminin after IGF-1 stimulation [58]. In addition, a recent study described that IRSs form high-molecular-mass complexes with a variety of proteins in a phospho-tyrosine-independent manner and modulate their availability to the IGF-1R [59, 60].

The second major pathway is through Shc, which reaches its maximal phosphorylation within 5–10 min of IGF-1R stimulation. Shc has been shown to consist of four unique members, ShcA, B, C, and D, and multiple splicing isoforms [61]. In general, Shc proteins possess a PTB domain at the N-terminal region and a SH2 domain at the C-terminal region. Between the PTB domain and SH2 domain, there are three tyrosine residues, possibly phosphorylated by IGF-1R, involved in Grb2 recruitment.

### PI 3-kinase pathway

One of the downstream pathways activated by the IGF-1R involves IRS interaction with a p85 regulatory subunit of PI3K class I, leading to activation of the catalytic subunit p110 of PI3K and inducing phospholipid products activating the downstream signaling pathway [62].

It has also been shown that tyrosine phosphorylation of the IGF-1R (Y1316XXM) can also induce direct binding of PI3K to the cytoplasmic region of the receptor [63] (Figs. 1, 2).

One of the main functions of PI3K is to synthesize the second messenger phosphatidyl inositol (3, 4, 5) triphosphate (PIP3) at the inner side of the membrane. These phospholipids function as ligands for recruiting PH domain-containing-proteins to the inner surface of the cell membrane [39]. The Akt/PKB serine threonine kinase interacts with these phospholipids causing its translocation to the inner membrane and activation by the 3-phosphoinositide-dependent protein kinases (PDKs) located around the membrane. IGFs-mediated activation of the PI3K pathway induces phosphorylation of the Thr308 and Ser473 residues on Akt and activates this kinase [64, 65]. Active Akt in turn phosphorylates and inhibits several pro-apoptotic proteins such as Bad [66] and caspase 9 [67], plus at least three other Akt effectors: the survival transcription factor cyclic AMP response element binding-protein (CREB), the pro-apoptotic effector protein glycogen synthase kinase-3β (GSK-3β), and the winged-helix family of forkhead transcription factors FKHRL1, FKHR, and AFX. Akt activation can also induce stimulation of mTOR that will lead to phosphorylation of the 40S ribosomal S6 protein by the p70S6 kinase, thereby enabling efficient translation of 5′ terminal oligopyrimidine tract (5′TOP) mRNA [68]. This class of mRNA is critically involved in the control of the protein synthesis machinery regulating the transition from G0 to G1 of the cell cycle. Activated mTOR can also induce phosphorylation of eukaryotic initiation factor 4E (eIF-4E) binding-protein (4E-BP), thus regulating cell cycle proteins like cyclin D1 [69]. mTOR is also involved in activation of matrix metallo proteinase (MMP)2 with effects on cell migration and metastasis potential [70]. Another effect of Akt activation is phosphorylation of Mdm2 on serine 166 and serine 186. Phosphorylation on these sites is necessary for translocation of Mdm2 from the cytoplasm into the nucleus, where Mdm2 decreases p53 transcriptional activity and hence diminishes cellular levels of p53 [71].

### MAP kinase pathway

The second major pathway is mediated by tyrosine-phosphorylated IRSs or Shc through another SH2-domain-containing signaling molecule, Grb2 (Figs. 1, 2). Grb2 is an adapter protein which possesses one SH2 and two SH3 domains. Grb2 can be activated by binding directly to phosphorylated IRS or Shc via its SH2 domain, while the SH3 domains interact with son of sevenless (Sos), a guanine nucleotide exchange protein. Sos stimulates the release of GDP and subsequent binding of GTP to the membrane-bound, low-molecular-mass GTP-binding-protein Ras. GTP-bound Ras interacts with and translocates the serine/threonine protein kinase Raf to the plasma membrane, where Raf becomes activated. Activated Raf phosphorylates and activates the dual specificity mitogen-activated protein kinase (MAPK) MEK, which in turn stimulates the ERK subset of MAPKs: extracellular-related kinase (ERK)1 and ERK2. ERK1/2 are translocated into the nucleus where they are involved in spindle formation. ERK also mediates transcriptional induction of the cyclin D1 gene, stimulating phosphorylation of the pRb protein and release of the E2F-1 transcription factor [72]. The free E2F-1 can activate in its turn the transcription of p14^ARF^ [73]. ARF was shown to inhibit the p53-Mdm2 association that maintains p53 in its inactive form [74]. In its turn, p53 can directly interfere with the Ras/MAPK cascade by inactivating ERK2/MAPK via caspase-mediated cleavage [75]. One of the other substrates of MAPK is p90Rsk. Both MAPK and p90Rsk translocate to the nucleus after phosphorylation, where they phosphorylate and activate transcription factors such as serum-responsive factor (SRF), T cell-specific transcription factor, and CREB, and, thus, alter gene expression. In the cytoplasm, MAPK regulates microtubule dynamics by phosphorylating microtubule-associated proteins. By regulating microtubule networks, MAPK regulates the CyclinB/Cdc2 complex: Cyclin B phosphorylation by MAPK is important for the translocation of the complex to the nucleus where it is activated by CDC 25C [76, 77].

### Other downstream signaling pathways

Previous studies to identify potential substrates of the IGF-1R and/or interactions with the receptor after its activation have revealed several additional possible downstream signaling molecules. One of these, Grb10, is a member of a super family of adaptor proteins, sharing a common overall structure, including an N-terminal region harboring a conserved proline-rich motif, a central PH domain, and a C-terminal SH2 domain. Ligand activation of the IGF-1R involves the Grb10 adaptor protein, which probably binds to autophosphorylated tyrosine residues located between amino acids 1229 and 1245 of IGF-1R [78] or tyrosine 1316 [79]. Activated Grb10 interacts with neuronal precursor cell-expressed developmentally downregulated 4 (Nedd4), and by doing this it has an important role in ubiquitination of the IGF-1R. Other substrates of the IGF-1R tyrosine kinase include the guanine nucleotide exchange factors Vav [80] and Vav3 [81], the adapters CrkII and CrkL [82], and focal adhesion kinase (FAK) [83]. Aside from IRS proteins, IGF-1R has been reported to interact in vitro and/or in vivo with numerous molecules, including Syp [39], GTPase-activating-protein [84], C-terminal Src kinase [85], and suppressor of cytokine signaling (SOCS)2 [86]. The IGF-1R can also activate the stress-activated protein kinases (SAPKs) including p38 and Jun N terminal kinase (JNK), a pathway associated with regulation of DNA damage responses and cell survival. It has been shown that RACK1, a small molecule that was identified as an IGF-1R interacting protein using a yeast two-hybrid interaction trap, regulates IGF-1R signaling and interaction of the IGF-1R with integrin signaling [87–89]. However, information regarding the roles of many of these molecules in IGF-1R signaling and biological functions is currently limited.

The classical IGF-1R signaling cascades are more complex, and they have been described in great detail in some dedicated reviews [1, 23, 33, 37, 41, 90–94].

## Something new: posttranslational modification of IGF-1R controlling IGF-1R expression and function

### Receptor internalization and signal attenuation

Owing to the presence of an intracellular tyrosine kinase domain, IGF-1R is classified as an RTK, and accordingly phosphorylation was considered to be the central process governing IGF-1R signaling [94, 95]. However, during the last decade, several laboratories have been investigating the mechanisms controlling the subsequent receptor downregulation and signaling desensitization. In this context, other posttranslational modifications including dephosphorylation, serine phosphorylation, ubiquitination, and sumoylation are increasingly recognized as modulating receptor levels and function.

Many cell surface receptors undergo endocytosis, being incorporated into clathrin- [96] or caveolin-coated vesicles. Some receptors are internalized constitutively and recycled (e.g., the transferrin receptor); however, with most RTKs and G-protein-coupled receptors (GPCRs), internalization is triggered by ligand binding [97, 98]. Ligand-activated receptors are normally downregulated by internalization [99–101], allowing the cells to return to an unstimulated, basal state. This internalization is considered to occur only for phosphorylated receptors [99–101], and is thus ligand-dependent.

Clathrin- or caveolin-mediated internalization then follows a process through early endosomes to late endosomes, during which decisions regarding fate of the proteins are made: whether to recycle or degrade. Early endosomes are mildly acidic, allowing detachment of the ligand and hence attenuation of the signal [102, 103]. If the receptor is to be degraded, it will progress to the highly acidic, hydrolase-containing lysosomal compartments. Internalization of a receptor does not necessarily mean immediate cessation of the signal followed by receptor degradation. This ligand-mediated endocytosis appears to have a double function: although subsequently attenuating the signal from activated receptors, it is also facilitating the interaction between the internalized receptor and the downstream signaling molecules [103]. For example, signaling through the EGFR is maintained in endosomal compartments and determines the longevity of the signaling [104, 105].

The ubiquitin pathway is a regulatory system for endocytosis [99–101, 106], and RTKs are well recognized as targets of ubiquitination [107]. Ubiquitination is the covalent attachment of the 7-kDa ubiquitin polypeptide to lysine residues on target proteins through the sequential actions of E1, E2, and E3 ligase enzymes [108, 109]. E1 and E2 work to load the E3 ligase with the ubiquitin, while E3 transfers the ubiquitin to the target protein. The E3 provides the specificity for the substrate, binding directly or through adaptor proteins. Ubiquitination can either be mono- (single ubiquitin moiety) or poly- (where chains of ubiquitin are added). Proteins can be multi-ubiquitinated (at multiple lysine residues) and polyubiquitination can be straight or branched type, depending on which of the lysine residues within ubiquitin the subsequent ubiquitin is attached [101, 110–112]. The type of ubiquitination is increasingly being recognized as determining the fate of the substrate protein [113]. Old or damaged cytosolic proteins are labeled with a polyubiquitin chain, which is recognized by the proteasome, multi-subunit proteolytic enzymes situated in the cytoplasm.

In addition to the degradation of cytosolic proteins, ubiquitin is also implicated in the internalization and degradation of plasma membrane proteins. In mammalian cells, a number of membrane proteins which are ubiquitinated are degraded through both the proteasome and lysosomal pathways [100], including the IGF-1R [114–117] and other RTKs [118, 119]. In some cases (e.g., the RTK Met), cytoplasmic fragments are cleaved from the receptor and degraded by the proteasome, in a complementary mechanism to lysosomal degradation [119].

### IGF-1R internalization, recycling, and degradation

The IGF-1R has been shown to be ubiquitinated [116, 117, 120] and internalized, through both clathrin and caveolin routes, in a ligand-dependent manner [116, 117, 120, 121]. Ubiquitination of the IGF-1R occurs prior to entry into endocytotic vesicles [120]. IGF-1R internalization reaches an apparent plateau after 20 min at 37 °C in cultured hippocampal neurons, while half-maximal IGF-1R internalization was obtained after 5 min at 37 °C in NIH-3T3 fibroblasts [122]. After internalization, some receptors are sorted for recycling to the cell surface. In activated T lymphocytes, internalization of the IGF-1R from the cell membrane was accompanied by a reduction in its mRNA. This was followed by re-expression of IGF-1R on the cell surface and an increase in IGF-1R mRNA levels in the cytoplasm, reaching levels higher than those initially recorded. However, a slower increase in the mRNA levels indicated that the earlier recovery of IGF-1R results from receptor recycling, followed by de novo synthesis [123]. There are certainly internal routes where the IGF-1R is recycled back to the cell surface and where this balance between recycling and degradation can be manipulated [124]. After internalization, IGF-1R degradation is mediated by both the proteasome and lysosomal pathways or recycled to the plasma membrane [100, 114, 116, 117, 120], although the relative roles of each are not clear.

A key question is: what is the downregulation signal for IGF-1R? To answer this question, at least two models have emerged during the last decade: one postulating internalization by a tyrosine-based motif and another by an ubiquitin-based motif. However, these two are not mutually exclusive, with phospho-tyrosine motifs potentially modulating ubiquitination and hence mediating receptor internalization.

### Tyrosine motif-based downregulation model

The ligand-activated receptors are targeted to clathrin-coated membrane invaginations [125], a process mediated by a specific internalization signal situated within cytoplasmic domain of the receptor [100]. There is evidence that internalization signals have a tyrosine-based motif usually located within the juxtamembrane region of the receptor [126, 127]. The human IGF-1R contains three tyrosine residues in the juxtamembrane region [50] that may be involved in internalization. However, contradictory results have been reported regarding the role of these tyrosine-based motifs as internalization signals. Prager et al. [50] demonstrated that the NPXY motif in IGF-1R is important for receptor internalization, whereas Miura et al. [128] demonstrated that tyrosine 1250 within the IGF-1R tail is the functional tyrosine-based internalization signal (Fig. 1).

### Ubiquitin motif-based downregulation model

Degradation of the IGF-1R by a proteasome mediated route was originally described by Sepp-Lorenzino et al. [114] to explain the mechanism of Herbymicin A-induced IGF-1R downregulation. Herbymicin A was demonstrated to only degrade IGF-1R in the presence of functional ubiquitin E1 ligase activity, and its action was prevented by proteasome inhibitors while being insensitive to lysosomal inhibitors. Since then, the IGF-1R has been demonstrated to be a substrate of three E3 ubiquitin ligases: Mdm2 [116], Nedd4 [120], and c-Cbl [117].

After Grb10 recognition as an IGF-1R interacting partner and negative regulator of IGF-1 signaling [78, 129], a yeast two-hybrid screen subsequently identified Grb10 as a binding partner of the Nedd4 E3 ligase [130]. Grb10 overexpression increased ligand-dependent IGF-1R ubiquitination, receptor internalization, and degradation. This ubiquitination did not occur with a catalytically inactive Nedd4 or with mutant Grb10 unable to bind Nedd4 [120]. This work identified Nedd4 as an ubiquitin E3 ligase and Grb10 as the key adaptor protein to recruit Nedd4 to the IGF-1R. Further work from the Morrione laboratory described Nedd4 ubiquitination of the IGF-1R as predominantly of multi-monoubiquitination type [131]. In addition, the internalization mediated by Nedd4 was both clathrin- and caveolin-dependent as demonstrated by co-localization studies [131].

Following original research demonstrating a feedback mechanism of wild-type p53 negatively regulating IGF-1R at the transcriptional level [132], it was revealed that overexpressed mutant or wild-type p53 mitigate ligand-induced IGF-1R downregulation [29]. Analysis of IGF-1R mRNA levels discard an exclusively transcriptional mechanism, indicating a posttranslational p53-IGF-1R control mechanism [29]. Further work demonstrated that an inhibition of p53 caused ubiquitination and degradation of the IGF-1R, suggesting that p53 and IGF-1R might compete for the same ubiquitin ligase [116]. This possibility was validated in experiments in which inhibition of Mdm2 (the well-known p53 ubiquitin ligase) resulted in accumulation of IGF-1R [116]. This work confirmed the direct IGF-1R/Mdm2 interaction, identified Mdm2 as an ubiquitin ligase for the IGF-1R, promoting proteasome-inhibitor-sensitive IGF-1R degradation, and highlighted a positive posttranslational control mechanism between p53 and IGF-1R [116]. Subsequent research revealed the mechanism of Mdm2 binding to the IGF-1R by identifying that β-arrestins, otherwise known as master regulators of GPCR biology, serve as adaptors to bring the E3 ligase Mdm2 to the IGF-1R [133]. Both Mdm2 and β-arrestin co-immunoprecipitated with the IGF-1R and in an in vitro reaction β-arrestins enhanced Mdm2-mediated IGF-1R ubiquitination [133]. In a cell system, overexpression or depletion of β-arrestin 1 enhanced and decreased IGF-1R ubiquitination and degradation, respectively. Thus, β-arrestin 1 was proved to act as an essential component in the ubiquitination and downregulation of the IGF-1R [133]. Most recently, β-arrestin 1 was recognized not only as an aid to IGF-1R internalization and signal cessation but to initiate its own second wave of signaling through the MAPK/ERK pathway [134], with IGF-1R stimulation also leading to ubiquitination of β arrestin 1 [134]. Intriguingly, this β-arrestin 1-mediated ERK activation occurs even when the classical IGF-1R kinase signaling is impaired (see below) [134]. Taken together, these studies demonstrated that β-arrestin 1 serves as an adaptor to bring the E3 ubiquitin-ligase Mdm2 to the IGF-1R, with a dual outcome on the IGF-1R: ubiquitination and receptor downregulation as well as IGF-1R/β-arrestin 1-mediated activation of MAPK signaling.

The identification of c-Cbl as an ubiquitin-ligase for IGF-1R [117] was based on the observation that suppression of Mdm2 did not completely abolish ligand-induced IGF-1R ubiquitination, suggesting that there are other ligases contributing to this process. Previously recognized as an E3 ligase of other RTKs, including EGFR and PDGFR [135], c-Cbl was demonstrated to associate with and to ubiquitinate the IGF-1R [117], especially at higher doses of IGF-1, indicating complementary roles for the different E3 ligases [117]. In vitro experiments using ubiquitin with mutated lysine residues suggested that Mdm2 polyubiquitinates IGF-1R with K63 type chains, whereas c-Cbl polyubiquitinates IGF-1R with K48 type chains. Co-localization between caveolin and an early endosome marker showed that higher doses of IGF-1 increased caveolin-mediated endocytosis, whereas low doses did not. In agreement with IGF-1 concentration determining ligase and endocytotic processes, overexpression of Mdm2 could reduce caveolin-mediated endocytosis.

The E3 ligases Mdm2 and Nedd4 have been demonstrated to bind to the IGF-1R through the adaptor proteins β-arrestin 1 [133] and Grb10 [131], respectively, suggesting that adaptor proteins determine substrate specificity. For Nedd4, there is evidence of the ligase determining appropriate localization of the receptor [136], and that it directs towards a proteasome-independent pathway of degradation [137]. The ligases c-Cbl and Mdm2 appear to have complementary roles, with c-Cbl recruited following high dose IGF-1 and initiating caveolin-dependent endocytosis and Mdm2 recruited at low dose IGF-1 and initiating clathrin-dependent endocytosis [117]. It is clear, however, that different ligases associated with the IGF-1R as well as their adaptor proteins have redundant as well as complementary roles: they keep the balance between recycling, relocalization, and proteasome/lysosomal degradation of the receptor [117, 120, 121, 131, 133, 134]. The complexity is further increased by the fact that receptor signaling relies on its localization: signaling still occurs from endosomes and prevention of endocytosis is even inhibitory to signaling [84, 124, 134, 138].

Through IGF-1R mutation analysis, the interplay between phosphorylation and ubiquitination has been demonstrated [121]. According to this study, a completely kinase-dead IGF-1R (with mutation of the ATP pocket) cannot be ubiquitinated after ligand stimulation, while a kinase impaired IGF-1R (Y1136A) is ubiquitinated, activates ERK signaling, but fails to phosphorylate Akt. Deletion of the C-terminal tail (Δ1245) had no effect on IGF-1R phosphorylation but completely abolished its ubiquitination [121], confirming the previous findings that IGF-1R ubiquitination is essentially dependent on its β-arrestin binding domain [133, 134].

Although initially described as a modification initiated by the natural ligand binding to the receptor, recent studies suggest that IGF-1R ubiquitination is a more complex process that can also be activated kinase-dependent (activation-loop phosphorylation) by IGF-2 [139], insulin [139], LL-37 [140], or kinase-independent by anti-IGF-1R antibodies [140–142]. In addition, IGF-1R ubiquitination can be activated in a ligand and kinase-independent manner, from inside the cell, by adaptor proteins recruited to the intracellular domain of the IGF-1R [124, 143, 144]. The mechanism of IGF-1R ubiquitination is different depending on the ligand, adaptor protein, or ubiquitin-ligase employed; however, the common theme is that a specific receptor conformation may trigger ubiquitination with divergent effects on receptor signaling, trafficking, and biological outcomes. It should be noted here that such conformation-activating ubiquitination is not essentially dependent on ligand–receptor interaction or activation-loop phosphorylation.

### IGF-1R dephosphorylation

Direct IGF-1R dephosphorylation has been little investigated. Rocchi et al. [145] described the association of activated IGF-1R with the phosphatase SHP2, with binding between phospho-tyrosines on IGF-1R and SH2 domains in SHP2 being critical. Subsequently, it was demonstrated that SHPS2 is critical for recruitment of SHP2 to the plasma membrane for this purpose [146]. Cross-talk with the αVβ3 integrin, through SHP2, was later described, with blockage of this integrin reducing IGF-1-induced IGF-1R phosphorylation [146, 147]. The phosphatase PTP1B also negatively regulates the IGF-1R [148], associating with it in a ligand-dependent manner [149].

### Nuclear IGF-1R

Recently, there has been greater appreciation of plasma membrane receptor relocalization to the nucleus. Nuclear localization has been demonstrated for the IGF-1R and shown to be mediated by sumoylation, the addition of a small protein similar to ubiquitin [150, 151]. The direction to the nuclear compartment and the role of nuclear IGF-1R is currently little investigated, but studies suggesting that IGF-1R nuclear localization can alter transcription [152], and that nuclear localization of IGF-1R predicts better outcome in patients treated with IGF-1R antibody [153], suggest that further research would have therapeutic relevance. One study has demonstrated that transfer to the nucleus is mediated at least in part by clathrin, and confirmed that both the α- and β-chains of IGF-1R translocate to the nucleus [154], suggesting a more complex function to IGF-1R internalization than solely degradation versus recycling. The emerging role of nuclear IGF-1R has recently been thoroughly reviewed by Sarfstein and Werner [155].

## Something borrowed: IGF-1R transactivation by other plasma-membrane receptors

It is generally accepted that several posttranslational modifications control the IGF-1R levels at the cell surface, with direct impact on receptor signaling. Are these modifications, in combination with the “two-states” model, sufficient to explain the differential signaling efficacy of the IGF-1R? Described by terms such as “cross-talk” or “transactivation”, it is increasingly recognised that IGF-1R signaling and efficacy is modified by interactions with other plasma-membrane molecules. This group include other RTKs, integrins, and GPCRs. Among the RTKs, probably the best studied is the IGF-1R interaction with the IR, resulting in formation of hybrid receptors [187, 188] which are able to respond to insulin, IGF-1, and IGF-2 but with different affinities. The mechanism of hybrid receptor activation, signaling, as well as their biological effects, have been extensively investigated [37, 139, 156–158]; however, it should be highlighted here that simple interaction of the IGF-1R with IR modified IGF-1R signaling and trafficking to various ligands [139] and anti-IGF-1R antibodies [156]. The IR is not the only RTK associated with the IGF-1R; for example, a direct interaction between the IGF-1R and EGFR was identified in cancer cells, with EGFR depletion affecting IGF-1R ubiquitination, degradation, and signaling [159]. In a similar manner, another group of plasma-membrane molecules were demonstrated to modify the IGF-1R response to IGF-1. Integrins, as transmembrane receptors, transfer information from the extracellular matrix (ECM) to signaling pathways inside cells (outside–in signaling) or from within the cell (inside–out signaling) [160]. They form part of cell surface signaling complexes known as focal adhesions which link the cytoskeleton to the ECM and are intimately linked with cell adhesion and migration [160]. There has been increasing recognition of the bi-directional cross-talk between integrins and the IGF-1R signaling pathway [161]: integrin activation modulating the IGF-1R signaling and vice versa. Studies identifying the overlap between IGF-1R and integrin signaling pathways from the Clemmons group demonstrated IGF-1R-dependent “transactivation” of αvβ3 integrin [162]. The mechanism was described as due to the recruitment and phosphorylation/dephosphorylation of adaptor proteins. IGF-1 stimulation causes phosphorylation of the transmembrane adaptor protein SHPS1 and subsequent SHP2 recruitment [146]. Along with SHP2, IGF-1R through IRS1 recruits the focal contact adaptor protein paxillin and FAK, which are involved in the turnover of focal contacts. IGF-1 stimulation leads to SHP2 dephosphorylation of paxillin and FAK, as part of an integrin deactivation mechanism crucial for migration [163]. In further evidence, the DOK1 protein, itself an IRS protein, brings SHP2 to the αvβ5 integrin [164]. IGF-1 is well known to initiate cell migration, IGF-1 triggers integrin activation and binding to ECM [165], and evidence showed that αvβ3 and αvβ5 are involved in the migratory/invasive IGF-1 response [165, 166].

However, there is also increasing recognition that the activation state of integrins alters IGF-1R signaling. The SHP2 protein is not only recruited to integrins and dephosphorylates paxillin and FAK but regulates IGF-1R dephosphorylation, curtailing the IGF-1R signaling response [146], identifying it as a common molecule between the integrin and IGF-1R signaling pathways. The Clemmons group also describe integrin modulation of the SHPS1-IGF-1R association, with activation and tyrosine phosphorylation of αvβ3 integrin determining the recruitment to the IGF-1R of SHPS1 and subsequently SHP2, and involving IAP [167, 168]. Overall, ligand occupancy of at least some integrins is required for a sustained IGF-1 response [169], and transmembrane integrin complexes including SHPS1 and IAP modulate IGF-1R signaling through determining SHP2 recruitment (reviewed in [170]), with subsequent effects on cell behavior [171].

The O’Connor group described a further common node between integrins and IGF-1R. The RACK1 scaffolding-protein, known to interact with β1 integrin, was identified as also binding IGF-1R affecting Shc/Grb2 downstream signaling [87, 172]. Further, the O’Conner group demonstrated that RACK1 associated mutually exclusively with phosphatase PP2A or β1 integrin, controlled by IGF-1 stimulation [173]. However, this complex differed between transformed and non-transformed cells, with only transformed cells showing direct RACK1–IGF-1R interaction [174].

Recent work by the Takada group posited an extracellular determinant of cross-talk between the two pathways, in agreement with the co-localization of IGF-1R and integrins together at focal adhesion complexes. They demonstrated direct binding of IGF-1 ligand to αvβ3 integrin and α6β4 integrin and direct effects on IGF-1R signaling [175], including sustaining of cells during anchorage independence [176] [177]. In their further work, an IGF-1 mutant unable to bind integrins but able to bind IGF-1R had a dominant negative effect on IGF-1R-mediated tumorigenesis in vivo [178], suggesting further functional effects of such ternary complexes.

Together, these data show that integrin activation is able to modify the phosphorylation and signaling from the IGF-1R and subsequently the biological outcomes. As mentioned before, in addition to RTKs and integrins, members of the GPCR family, can modify the IGF-1R responsiveness to different ligands.

## Something borrowed: IGF-1R utilize components of GPCR signaling

It is generally accepted that RTKs share signaling pathways with the larger class of the GPCRs [3, 179]. Over the last decades, at least two mechanisms of receptor cross-talk between RTKs and GPCRs have been described: receptor transactivation and RTK signaling through GPCR components.

RTK transactivation is typically explained as the GPCR-dependent increase in phosphorylation, kinase activity, and signaling of the RTK. One example is represented by the lysophosphatidic acid (LPA) transactivation of the EGFR [180]: LPA, a classical GPCR agonist, triggers EGFR auto-phosphorylation and MAPK signaling activation, effects that are sensitive to EGFR-kinase inhibitors or expression of EGFR kinase-defective mutants [180]. Although not completely understood, the mechanism of LPA-induced transactivation of EGF receptors is likely to involve the release of an EGFR ligand by a GPCR-activated metalloproteinase. Several other RTKs, including the ones for PDGF, VEGF, and NGF, were reported to be transactivated by GPCR agonists such as LPA, angiotensin, endothelin, and bradykinin (for review, see [179]). It is noteworthy that RTK transactivation by GPCRs required tyrosine phosphorylation and kinase activity of the growth factor receptor and is sensitive to inhibitors of receptor kinases [179, 181]. The IGF-1R is not an exception from this rule: transactivation and phosphorylation of the IGF-1R with subsequent MAPK activation was reported following thrombin stimulation of aortic smooth muscle cells [182]. Several other GPCR-agonists were described as IGF-1R transactivators, including neurotensin [183] and vasopressin [184].

The second mechanism used to explain RTK/GPCR cross-talk is the employment of the GPCR signaling components, G-proteins and β-arrestins, by the RTKs for their signaling activation. Characteristic of this type of signaling is the response to G-protein signaling inhibitors (e.g., pertussis toxin which uncouples Gαi from an activated receptor), G-protein sequestration [185], or β-arrestin downregulation [144, 186]. Most if not all RTKs, including PDGFR, EGFR, and VEGFR, utilize G-proteins as signaling mediators (for in depth reviews, see [3, 179, 181, 187]). In this context, it is worth mentioning that the members of the IR family were the first described to engage the G-proteins signaling: the IR signaling was demonstrated to be sensitive to pertussis toxin treatment [186], with subsequent decrease of the insulin-induced inhibition of adenylyl cyclase in isolated hepatocytes [188]. Several other studies have demonstrated the Gαi involvement in IR signaling and their effects on insulin signaling biological outcomes [186, 189]. Given the high structural similarities between IR and IGF-1R, it is not surprising that some G-proteins have also been identified to physically associate and mediate IGF-1R signaling. Almost 20 years ago, a study from Robert Lefkowitz’s laboratory reported that IGF-1R-dependent activation of the MAPK signaling pathway was inhibited by the Gαi-inhibitor pertussis toxin or by sequestration of G-protein βγ subunits by a peptide derived from GRK2 [185]. This study clearly demonstrated that IGF-1R signaling depends on heterotrimeric G-proteins containing the Gαi and Gβγ subunits. Consistent with these observations, subsequent studies demonstrated that, in rat neuronal cells or mouse fibroblasts, Gαi and Gβ subunits were associated with IGF-1R while Gαs was not associated with the IGF-1R in any cell type. More importantly, this study demonstrated the IGF-1 induced release of the Gβ subunits from the IGF-1R with no effect on IGF-1R/Gαi association, indicating a discrete pool of Gβγ subunits available for IGF-1R downstream signaling [190]. The IGF-1R/Gαi association was also reported in a separate study investigating the roles of heterotrimeric G-protein signaling components in insulin and IGF-1 signaling. In 3T3L1 adipocytes, in basal state, Gαi and Gβ were associated with the IGF-1R, while IGF-1 stimulation increased the IGF-1R/Gαi association, releasing the Gβ subunits [144].

While RTK transactivation by GPCRs has long been recognized and is today considered a “classical” signaling pathway, the complexity of the RTK/GPCR cross-talk was further demonstrated by recent findings of bidirectional cross-communication between RTKs and GPCRs (for in depth reviews, see [181, 187]). In these processes, the GPCR signaling is activated by RTKs by the same two mechanisms described above: production of a GPCR ligand stimulated by RTK activation or a ligand-independent cross-activation of the signaling network [181, 187]. In the case of the IGF-1R, such GPCR-transactivation has been demonstrated to be essential for migratory and pro-survival functions controlled by IGF-1 [181, 187]. Taken together, these studies indicate a common signaling platform between IGF-1R and GPCRs to differentiate between the responses upon combined growth factor/GPCR agonist stimulation from single stimulation by either ligand.

The corollary of these studies is that IGF-1R (and IR) can activate signaling as a GPCR, using different G-protein partners for downstream signaling. The Gαi subunit is constitutively associated with the IGF-1R while IGF-1 treatment leads to GTP loading of Gαi2 [144] and Gβγ dissociation. Yet, these findings raise another critical question: if IGF-1R and IR can activate G-protein signaling, what is the system desensitizing this pathway? In the case of GPCRs, the signaling is terminated by β-arrestin recruitment to the activated receptor. Early studies demonstrated the involvement of β-arrestin in IGF-1R signaling and trafficking: a dominant negative mutant of β-arrestin 1 was demonstrated to impair IGF-1R internalization, whereas overexpression of wild-type β-arrestin 1 or β-arrestin 2 increases IGF-1R internalization [191]. Moreover, G-protein signaling activated by IGF-1R was demonstrated to be sensitive to β-arrestin inhibition [144].

The discovery of the dual regulatory role of β-arrestin 1 in the case of IGF-1R, downregulation [133], and signaling activation [134], pointed towards a remarkable parallel with the role of β-arrestins in the case of the larger GPCR family. While internalizing the GPCR and ending G-protein signaling, β-arrestins activate the MAPK pathway [192–194].

Recognized as a universal mechanism of GPCR regulation, β-arrestin 1 binds to the receptor and desensitizes G-protein signaling only after phosphorylation of specific serine residues by the G-protein–coupled receptor kinases (GRKs) [193, 195, 196]. Therefore, a legitimate question was whether the same mechanism is in place for the IGF-1R.

Investigating this scenario, we found that activated IGF-1R allows recruitment of the GRK proteins, specifically with contrasting effects between GRK2 and GRK6 [124]. Subsequent GRK2- or GRK6-dependent phosphorylation of IGF-1R C-terminal serine residues 1248 or 1291, respectively, allows β-arrestin 1 recruitment, with the residue that is phosphorylated controlling the duration and strength of the β-arrestin/IGF-1R association.

**Fig. 3:**
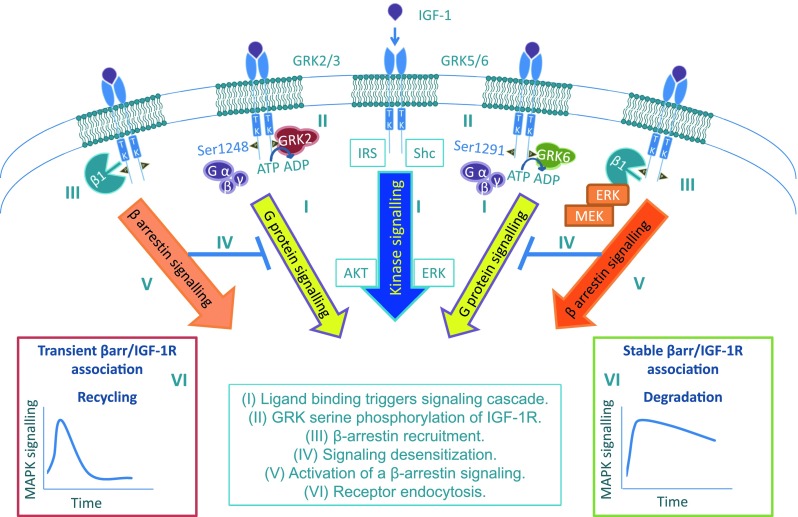
IGF-1R as a RTK/GPCR functional hybrid. Signaling: agonist-stimulation of the IGF-1R triggers the classical RTK signaling leading to downstream kinase-cascade signaling activation. In addition, agonist-stimulation of IGF-1R leads to non-canonical GPCR signaling through heterotrimeric G-proteins (G α, β, γ) (*I*), following which the receptors are rapidly phosphorylated by G-protein-coupled receptor kinases (GRKs) at serine residues within the C-terminus (*II*). Desensitization: Serine-phosphorylated receptors present high affinity binding sites to recruit the multifunctional adaptor protein β-arrestin 1 (β1) (*III*). Steric binding by β-arrestin to the IGF-1R C-terminal prevents further G-protein coupling, leading to the desensitization of G-protein-dependent signaling (*IV*). β-arrestin signaling: β-arrestin acquires an active conformation upon binding the IGF-1R and scaffolds components of the MAPK pathways leading to the activation of a second wave of IGF-1R kinase independent signaling through β-arrestin 1 (*V*). The dynamics of this signaling activation is determined by the strength of the β-arrestin 1/IGF-1R interaction, dependent on GRK-isoform (*VI*) Such β-arrestin-dependent MAPK activity has been shown to regulate multiple IGF-1R biological effects including proliferation, apoptosis, cell migration, and cancer metastasis. Endocytosis: agonist-stimulation promotes rapid endocytosis of the IGF-1R. This internalization is enabled by β-arrestin binding, connecting the receptor to the endocytic machinery efficiently. The interaction between β-arrestin and the E3 ubiquitin ligase Mdm2 promotes IGF-1R and β-arrestin ubiquitination that facilitates IGF-1R endocytosis, followed by post-endocytic sorting of internalized IGF-1R. The strength of the β-arrestin 1/IGF-1R interaction GRKs-isoform-dependent determines the fate of internalized receptor: recycling (transient β-arrestin 1 binding) or degradation (sustained β-arrestin 1 binding) (*VI*)

Identification of GRK-dependent phosphorylation of IGF-1R serine residues as the underlying mechanism for β-arrestin/IGF-1R interaction not only revealed another connection to the complex cross-talk between the IGF-1R and GPCR but in fact provided the missing link to functionally identify IGF-1R as a GPCR. This paradigm shift is founded on the accepted universal model of GPCR activation and desensitization delineated by six distinct processes [195, 197], all of which have been identified to occur for the IGF-1R (Fig. 3): (1) ligand binding to the IGF-1R, in addition to the classical kinase signaling cascade triggers signaling through heterotrimeric G-proteins [144, 185], (2) subsequent GRK2- or GRK6-dependent phosphorylation of IGF-1R C-terminal serine residues 1248 or 1291, allowing β-arrestin binding to these specific phosphorylated serine residues [124], with (3) β-arrestin recruitment [124, 133, 134], (4) subsequent kinase/G-protein signaling desensitization, (5) activation of a β-arrestin-dependent second signaling wave through MAPK [124, 134], and (6) receptor endocytosis with the GRK isoform determining receptor degradation [124, 133] or recycling [124]. In this model, one uncertainty is whether, for the IGF-1R, GRK-mediated β-arrestin binding initiates a desensitization process (Fig. 3, IV) and whether the desensitization process affects both G-protein and kinase signaling. While for GPCRs the key role of GRKs in desensitization is well recognized, for RTKs few studies have investigated the contribution of GRKs [3]. In all published studies, GRK2 is reported to desensitize or modulate RTK signaling. For instance, GRK2 is recruited and co-localizes with the ligand-activated EGFR or PDGFR, leading to receptor serine phosphorylation and increased ERK activation in the case of EGFR [198], or inhibiting the kinase activity in the case of PDGFR [199]. The insulin receptor (IR), closely related to the IGF-1R, is a special case: GRK2 was shown to have inhibitory effects on IR-mediated signaling and glucose uptake, though the observed effects were demonstrated to be mediated in a kinase-independent manner through GRK2 sequestration of Gαq/11 [200].

For GPCRs, the desensitization requires the membrane localization of GRKs. In a palmitoylated state, GRK6 has been found to be exclusively associated with the membrane; however, it is not yet clear whether this reversible posttranslational modification is induced by activated receptor [3]. On the other hand, a clear regulatory negative feedback, induced by activated receptor, was demonstrated for GRK2: Gβγ subunits, generated by the agonist-occupied receptor, interact with GRK2 and serve to target this enzyme into proximity with its membrane receptor substrate [201]. Consequently, β-arrestin recruitment to GRK-phosphorylated receptors physically prevents the coupling of receptor to its cognate G-protein. Thus, a true comparison of IGF-1R desensitization with GPCR desensitization would make sense only if IGF-1R utilizes heterotrimeric G-proteins for its signaling. Our results showing that GRK2 is involved in limiting the ERK response to IGF-1 fully support the GRK2 desensitization function in the case of IGF-1R. For GRK6, our interpretation is that prolonged ERK activation is due to the second wave of signaling activated by the stable β-arrestin recruitment to the IGF-1R (Fig. 3, V), masking the desensitization of the first signaling wave. Taken together, the proposed model represents IGF-1R as not only “borrowing” components of GPCR signaling but to operate as a functional hybrid RTK/GPCR (Fig. 3).

## The emerging paradigm for IGF-1R signaling

A major limitation of the two-state model for IGF-1R signaling is the explanation of the two main components of the IGF-1R activity: the signaling activation and receptor downregulation as separate events. In this paradigm, all receptors are equal and the receptor activity is exclusively and directly related to kinase activation by ligand–receptor interaction. Signaling, and downregulation triggered simultaneously by the ligand-activated receptor. Both involve phosphorylation, ubiquitination, and probably other posttranslational modifications. The logical conclusion is that receptor signaling and degradation never dissociate from each other or from ligand-induced receptor (kinase) activation. Moreover, in the same cellular context, the MAPK/ERK and PI3K/Akt pathways are described as being activated in a balanced manner following IGF-1R stimulation. Yet, there is experimental data that do not support this model, including some major contradictions (Table 2) such as kinase-independent signaling activation [124, 134, 202] or unbalanced signaling in the same cellular background [34, 124, 134], signaling-degradation dissociation, or receptor downregulation in the absence of the ligand or activation-loop phosphorylation [37, 124, 203]. Beyond this, there is the major paradox of the IGF-1R inhibitors (antibodies and small molecules inhibitors, see below), which are able to activate IGF-1R downregulation and signaling despite clear inhibitory effects on receptor tyrosine phosphorylation.

**Table 2:** The new paradigm for IGF-1R signaling

Classical paradigm and its contradictions	Emerging paradigm
Receptor activity is exclusively and directly related to kinase activation by ligand–receptor interaction **Contradiction**: kinase-independent signaling activation: IGF-1R can trigger MAPK signaling in the absence of the ligand or without kinase domain activation [124, 134, 202]	In addition to classical RTK signaling, IGF-1R operates as a functional GPCR In this model, IGF-1R can initiate kinase-independent signaling such as β-arrestin signaling and heterotrimeric G-protein signaling
Signaling and receptor downregulation are triggered simultaneously by the ligand-activated receptor in a balanced manner **Contradictions**: signaling–downregulation dissociation-specific point-mutations outside the kinase domain can activate signaling or receptor downregulation in the absence of the ligand or activation-loop phosphorylation [124] IGF-1R downregulation induced by ligands other than IGF-1 (e.g., antibodies) Unbalanced signaling downregulation induced by IGF-1R transactivation (e.g., hybrid receptors, integrins, other GPCR or RTKs)	The receptor conformation activating the kinase signaling can be distinct from that which interacts with β-arrestins facilitating receptor ubiquitination and endocytosis in the absence of kinase activity This model can explain the dissociation between kinase activation and receptor degradation as well as ligand- or kinase-independent signaling or receptor downregulation triggered by kinase inhibitors (e.g., targeting antibodies) or IGF-1R partners
Equal receptors with balanced activation of the downstream signaling pathways (MAPK/ERK and PI3K/Akt pathways) **Contradictions**: unbalanced signaling: in the same cellular background, IGF-1R can preferentially activate either the MAPK or PI3K/AKT pathways [34, 124, 134] The paradox of the IGF-1R inhibitors (antibodies and small molecules inhibitors) which are able to activate IGF-1R signaling despite clear inhibitory effects on receptor tyrosine phosphorylation	The receptor conformation activating the kinase signaling can be distinct from that which interacts with β-arrestins or with other partners In this model, not all receptors are equal and their activity can be modulated from inside the cell by particular posttranslational modifications (e.g., serine phosphorylation, ubiquitination, etc.) or by interacting proteins (e.g., β-arrestins, IR, integrins, etc.) The same model would also accommodate the unbalanced IGF-1R signaling, activated in a “biased manner” via β-arrestin by IGF-1R inhibitors as well as by natural “biased” agonists

The contradictory evidence against the two-state model is described and how the new paradigm can explain these data

**Fig. 4:**
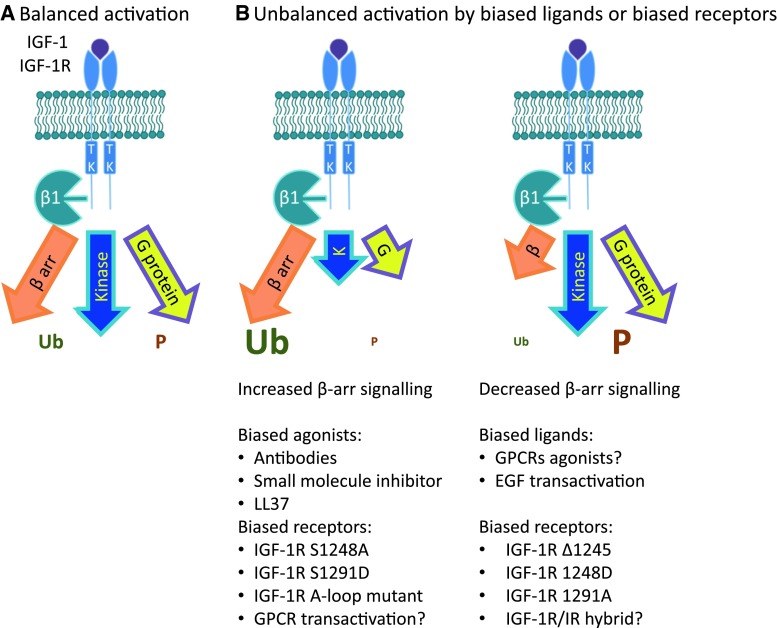
New and old paradigms of IGF-1R signaling: balanced and biased signaling at the IGF-1R. **a** Classical model: IGF-1R activation is triggered exclusively by ligand-binding and signaling is mediated by kinase cascade through phosphorylation. Ligand-activated IGF-1R leads to balanced phosphorylation-dependent Akt/ERK signaling and balanced signaling/downregulation. **b** New paradigm. In the current model for IGF-1R activation, in addition to the classical RTK-cascade, binding of a ligand results in activation of signaling by G-proteins and β-arrestins, as well as desensitization and internalization by β-arrestins. In a system with balanced signaling, ligand-binding results in balanced activation of kinase signaling, signaling by G-proteins and β-arrestins, as well as desensitization and internalization by β-arrestin. In a system with biased signaling, two alternative outcomes of receptor activation are depicted: kinase/G-protein-biased with enhanced IGF-1R phosphorylation and β-arrestin-biased with enhanced IGF-1R ubiquitination. Biased signaling is a feature of the ligand–receptor complex so either the receptor or the ligand could be biased. An IGF-1R biased ligand elicits one response over another compared with the classical ligand, (e.g., anti-IGF-1R favors β-arrestin signaling). A biased IGF-1R is only efficient at activating a restricted subset of downstream signaling pathways (e.g., mutant receptors)

The appreciation of the dual functions of β-arrestin, as a mediator of IGF-1R signaling [124, 134, 191, 204] as well as mediator of receptor downregulation [124, 133], provide the basis for the emerging paradigm of IGF-1R signaling. In this model, IGF-1R can initiate classical kinase signaling, β-arrestin signaling, and heterotrimeric G-protein signaling as well as β-arrestin-mediated receptor desensitization (Fig. 4). However, the receptor conformation activating the kinase signaling can be distinct from that which interacts with β-arrestins, as demonstrated by the IGF-1R mutants constitutively binding β-arrestin, that are degraded even in the absence of the ligand [124] (Fig. 4). This scenario can explain the dissociation between kinase activation and receptor degradation as well as kinase-independent signaling. The same model would also accommodate the unbalanced IGF-1R signaling, activated in a “biased manner” via β-arrestin by IGF-1R inhibitors as well as by natural “biased” agonists [22, 143]. In this emerging model, not all receptors are equal and their activity can be modulated from inside the cell by particular posttranslational modifications (e.g., serine phosphorylation, ubiquitination, etc.) or by interacting proteins (e.g., β-arrestins, integrins, other RTKs).

## Implications for treatment

Owing to its essential role in maintaining the malignant phenotype, IGF-1R-targeted therapy was considered a very promising strategy for cancer treatment [205–214]. Although several approaches targeting the IGF-1R were employed to prove the concept, the common aim for all these strategies is the inhibition of the classical kinase signaling cascade (for reviews, see [26, 27, 91, 215]). This can be accomplished either by preventing ligand–receptor interaction (e.g., using blocking antibodies) or silencing the effects of this interaction (e.g., tyrosine kinase inhibitors, TKIs).

### Strategies aimed at blocking the ligand–receptor interaction

Receptor downregulation was among the first approaches used to prevent ligand–receptor interaction and to validate IGF-1R as a therapeutic target [216]. This was achieved by antisense oligonucleotides, plasmids expressing IGF-1R antisense cDNA or triple helix-forming oligodeoxynucleotides [23]. The same outcome was obtained with drugs interfering with IGF-1R glycosylation, folding and expression at the cell surface (tunicamycin or lovastatin) [17–19, 217] or by directly increasing receptor degradation [114, 218]. The majority of these systems were unsuitable for transfer to the clinic, either because they were unable to be delivered or because they were too unspecific, so the next work in this area focused on clinically suitable methods for blocking the ligand–receptor interaction.

### Competition with the ligand–receptor interaction

In this category are included strategies that involve upregulation of the IGFBPs, the natural IGF inhibitors [219], IGFs peptide analogues [219, 220], or ligand or receptor neutralizing antibodies [206, 221]. There are seven different IGFBPs, most of them have been shown to inhibit the actions of IGFs. In vitro and in vivo experiments indicate that increasing IGFBPs could be an alternative to IGF-1R targeting, particularly due to the lack of interference with insulin signaling [222]. There is also evidence that free IGFBPs have anti-tumor activity independent of their IGF-binding capacity [223]. Anti-ligand antibodies, developed to mimic IGFBP action, have a high affinity against both IGF-1 and IGF-2, and do not cross-react with insulin [224–226]. Several antibodies targeting IGF-1 or IGF-2 have been reported, with one reaching clinical trial [225]. They prevent signaling through the IGF-1R and the hybrid receptors, but, importantly, they do not affect insulin-stimulated phosphorylation of the insulin receptor (IR) and its downstream signaling. While most of the ligand-targeting antibodies bind to both IGFs, a few have higher affinity for IGF-2 [224, 226, 227]. Notably, antibody sequestration of ligand can lead to high levels of free IGFBPs, a consequence that might improve their intended effect [93].

Antibodies against the extracellular ligand binding domain of the IGF-1R were designed to block ligand–receptor interaction [27, 93]. This strategy was considered to be very promising, and therefore most of the large pharmaceutical companies developed anti-IGF-1R antibodies and those demonstrating significant activity in preclinical settings were taken forward for clinical evaluation [27, 93]. However, in clinical settings, treatment with anti-IGF-1R antibodies induced clinical responses only in some cases of Ewing’s sarcoma (ES) [228–230] and selected cases of lung carcinoma [93, 231, 232]. Although not chosen by design, all anti-IGF-1R antibodies used in clinical trials, besides inhibiting IGF-1-induced receptor phosphorylation, also trigger receptor downregulation ([140] and below).

### Strategies aiming to inhibit IGF-1R signaling

The proof of mechanism for this approach is represented by IGF-1R kinase-dominant negative mutants, successfully tested in preclinical settings by several laboratories [211, 233]. Cells transfected to express IGF-1R cDNA with kinase-inactivating [234] or kinase-limiting mutations [235, 236], lose their malignant phenotype as well as their invasive and metastatic potential [237]. The promising strategy of inhibiting IGF-1R kinase activity is complicated by IR cross-reactivity issues, as the kinase domain of the IGF-1R shares 85 % homology with that of the IR, with the ATP binding cleft 100 % conserved [40]. Nevertheless, several small inhibitors have been developed (for review [238, 239]) and despite some of them having shown signs of cross-reactivity with the IR, they are still considered for use in clinical settings. Additionally, there is recent evidence and debate in the literature as to whether co-inhibition of the IR is beneficial for anti-IGF-1R therapy [240, 241]. While all small molecules targeting IGF-1R prevent kinase activation, they can be further divided in two subgroups, based on their mechanism of action: inhibitors of the ATP binding cleft or inhibitors of the kinase–substrate interaction, including inhibitors of the A-loop. Among the first category, NVP-AEW541 and NVP-ADW74 inhibit IGF-1R kinase activity and appear to be equipotent for both IGF-1R and IR inhibition in vitro, yet they show selectivity for the IGF-1R in intact cells. The two drugs were tested in various models such as fibrosarcoma, myeloma, and ES [242, 243]. Another small molecule, OSI-906 is a dual IGF-1R/IR kinase inhibitor with a strong anti-tumoral efficiency in an IGF-IR-driven xenograft model [244]. There have been several such inhibitors tested in clinical trials [27], and what they have in common is that inhibition of the ATP-binding also prevents IGF-1R downregulation. This is not surprising as in vitro experiments clearly demonstrated that IGF-1R ATP-defective mutants are not ubiquitinated [121].

Among the second category of the IGF-1R kinase inhibitors (TKIs), the cyclolignan picropodophyllin (PPP) demonstrates a special feature for this class. PPP was originally described to inhibit the IGF-1R without altering the IR, reducing Akt phosphorylation [20] and inducing tumor regression in xenografted mice. Rather than competing with ATP, PPP differs from other IGF-1R TKIs by interfering with phosphorylation of the kinase domain A-loop. PPP specifically blocked phosphorylation of the tyrosine (Y) 1136 residue, while sparing the two others (Y1131 and Y1135), suggesting that it might act as a substrate inhibitor [245]. Since its discovery [20], PPP has been proven to inhibit IGF-1R biological activities in a very large number of experimental models as well as in clinical settings [14, 16, 24, 90, 246–258], has been demonstrated to produce very limited resistance [22, 259, 260] to anti-IGF-1R therapy, and has proven efficacy in clinical trials [261]. The remarkable feature of PPP that differentiates it from ATP-inhibitors is that, similar to anti-IGF-1R antibodies, PPP also triggers IGF-1R downregulation [143]. This feature deserves special attention because it supports the logical but still contentious [90] assumption that receptor downregulation is desirable in addition to inhibition of its TK activity [15, 95].

Yet, the debate on how important receptor downregulation is in relation to kinase inhibition is hiding a paradox: how is the receptor downregulated by inhibiting its kinase activity?

The classical, binary ON/OFF model for IGF-1R signaling and degradation implies that the only way to maintain the receptor in an ‘OFF’ state is by preventing kinase activity while degradation would only occur with an ‘ON’ state receptor. Evidently this model is insufficient, because it cannot account for the contradiction that anti-IGF-1R antibodies (or PPP) trigger receptor degradation in the absence of kinase activation. This contradiction was recently investigated for the case of anti-IGF-1R antibody (Figitumumab)-induced receptor degradation [140]. Although the antibodies were primarily designed to block the ligand–receptor interaction thus preventing kinase activity, it has been demonstrated that targeting antibodies act as “biased” IGF-1R agonists, activating β-arrestin recruitment to the receptor, with subsequent receptor ubiquitination, internalization, ERK signaling activation, and IGF-1R degradation [140]. In agreement with this, anti-IGF-1R therapy efficacy was proven to be highly dependent on β-arrestin 1 expression and modulated by co-targeting β-arrestin 1-mediated signaling [140]. Notably, a similar mechanism of β-arrestin 1-mediated receptor ubiquitination and downregulation with activation of the β-arrestin 1/ERK second signaling wave has also been demonstrated in the case of the PPP paradox [143].

The experimental data provided by these studies fully substantiate and support the new paradigm for IGF-1R signaling which allows for unbalanced signaling and kinase activation/receptor downregulation dichotomy (Fig. 4). According to this model, the conformation of IGF-1R, which activates the kinase cascade, can be distinct from that which interacts with β-arrestins or other signaling molecules. Receptor modification induced by ligands, small molecule inhibitors, or intra-cellular interacting proteins can further be interpreted as alterations to the IGF-1R conformation, there being more than just two (ON/OFF) conformations. Different conformations can be mutually exclusive and can determine the quality, quantity, and duration of output signaling from the receptor as well as the fate of the receptor (Fig. 4). Recognizing the dual role of β-arrestin in controlling receptor downregulation as well as kinase-independent signaling, the new model we propose (Fig. 4) can accommodate all experimental data, demonstrating that IGF-1R signaling is not exclusively dependent on its kinase activity and can be activated and downregulated in a “biased” manner via β-arrestin 1 by IGF-1R inhibitors or by natural “biased” agonists [143, 253] (Table 2). In the emerging model of IGF-1R signaling, the question of “how important receptor is downregulation in relation to kinase inhibition?” is getting a new meaning: “Is it possible to downregulate the IGF-1R without activating signaling?” As downregulation without signaling has never been recognized, a simpler solution would be to identify the biased signaling pathways and target them separately [140].

Although oversimplified and insufficient to explain several outcomes of IGF-1R activation, the classical paradigm is currently in use when selecting the agents targeting the IGF-1R. The best example is the case of anti-IGF-1R antibody: during their development, antibodies were designed to achieve the maximum binding to IGF-1R, to compete with the natural agonists [140]. In the subsequent drug screening for clonal selection, the assays were limited to confirmation of their inhibitory effects on IGF-1-induced receptor signaling [142, 262–265]. As long as the canonical IGF-1R model does not acknowledge kinase-independent signaling, it is not surprising that targeting antibodies were rarely evaluated in IGF-1-independent conditions to estimate their intrinsic agonistic potential: most measuring receptor phosphorylation, a few checking IRS/Akt activation, and none checking MAPK/ERK activity.

Despite big expectations regarding the use of anti-IGF-1R for cancer treatment, almost all clinical trials were stopped due to futility, whereas some pharmaceutical companies closed their programs for developing IGF-1R inhibitors [93]. Furthermore, the value of IGF-1R as a target for cancer therapy has been questioned [90, 93]. While several reasons for the failure of the anti-IGF-1R have already been discussed in detail [90, 93], the new paradigm we propose for the IGF-1R signaling highlights another potential cause for these unsatisfactory results: biased-signaling activation by anti-IGF-1R antibodies. The best example is again Figitumumab, designed to prevent the IGF-1 binding to its receptor and kinase-dependent signaling. In Ewing sarcoma, at least two resistance mechanisms have been reported, developed after Figitumumab treatment: formation of the IGF-1R/IR hybrid receptors [266] and biased IGF-1R signaling activation [140]. As described before, the IGF-1R/IR hybrid formation could be appreciated as a form of receptor bias. Challenging the classical two-state model in which the antibodies were designed, the new paradigm in which a biased-receptor (IGF-1R/IR hybrid) is activated by a biased-agonist (Figitumumab) provides an explanation as to why such a strategy did not work in clinical settings and advocates recognition of IGF-1R as a key target for cancer therapy and a clear choice for the baby rather than the bathwater [91].

## Concluding remarks

Today, targeting the IGF-IR and components of its signaling pathway in different forms of cancer is a major research area. Yet, the design of such targeting agents is based mostly on the classical ON/OFF model of IGF-1R activation. The present review highlights the fact that, in addition to the classical kinase pathway, IGF-1R activity and its biological effects are controlled by a variety of adaptor/signaling-proteins through IGF-1R posttranslational modifications, including tyrosine and serine phosphorylation, de-phosphorylation, ubiquitination, and sumoylation. The complexity of IGF-1R behavior following exposure to IGF-1R inhibitors reinforces the need to understand the relationships between different signaling pathways and between signaling and degradation. Only this can lead to the rational design and testing of IGF-1R inhibitors and the successful combining of these therapeutics with others targeting parallel pathways. Searching for therapies inhibiting only IGF-1R kinase activity may appear to invalidate the IGF-1R as a target for cancer therapy, while many potential drugs that modify alternative downstream effects, the “biasing agonists”, are not considered.

## Abbreviations

aa: Amino acid residues
A-loop: Activation loop
ATP: Adenosine triphosphate
CH2: Collagen homology 2 domain
C-terminal: Carboxyl terminal
cAMP: Cyclic adenosine monophosphate
CREB: cAMP response element binding-protein
ECM: Extra-cellular matrix
EGFR: Epidermal growth factor receptor
ERK: Extracellular-related kinase
ES: Ewing sarcoma
GDP: Guanosine diphosphate
GSK 3β: Glycogen synthase kinase
GTP: Guanosine triphosphate
G proteins: Guanine nucleotide binding-proteins
GPCRs: G-protein coupled receptors
Grb2: Growth factor receptor bound protein
GRKs: GPCR-related kinases
IGF: Insulin-like growth factor
IGFBP: Insulin-like growth factor binding-protein
IGF-1R: Insulin-like growth factor-type 1 receptor
IR: Insulin receptor
IRR: Insulin receptor-related receptor
IRS: Insulin receptor substrate
MAPK: Mitogen-activated protein kinase
Mdm2: Mouse double minute 2 homolog
MEK: Mitogen-activated protein kinase
N-terminal: Amino terminal
PDK: 3-Phosphoinositide-dependent protein kinase
PH: Pleckstrin homology
PTB: Phosphotyrosine binding domain
PI3K: Phosphoinositol 3 kinase
PIP3: Phosphoinositol triphosphate
PPP: Picropodophyllin
RTKs: Receptor tyrosine kinases
TK: Tyrosine kinase
TKI: Tyrosine kinase inhibitor
SAPK: Stress-activated protein kinase
SH2/3: Src homology 2/3 domain
Shc: SH2 domain-containing-protein
SOCS: Suppressor of cytokine signaling
Sos: Son of sevenless
SRF: Serum responsive factor
TKI: Tyrosine kinase inhibitor
